# Characterization of *Helicobacter pylori* aggregation reveals a requirement for both AlpA and AlpB

**DOI:** 10.1128/jb.00264-26

**Published:** 2026-06-22

**Authors:** Alexander Solov, Xiaolin Liu, Yasveck Duran-Ramirez, Corey Witt, Karen M. Ottemann

**Affiliations:** 1 Department of Microbiology and Environmental Toxicology, UC Santa Cruz 542823 , Santa Cruz, California, USA; National Institutes of Health, Bethesda, Maryland, Bethesda, USA

**Keywords:** aggregation, biofilms, outer membrane proteins

## Abstract

*Helicobacter pylori* is a gram-negative human pathogen that colonizes the stomach and is a major risk factor for gastric ulcers and cancer. A common but poorly understood characteristic of *H. pylori* is its propensity to aggregate in liquid culture. To investigate this phenomenon, we developed protocols to form, measure, and disperse aggregates. Using these tools, we determined that *H. pylori* aggregation is protein-dependent and can occur non-clonally, from the binding of distinct cells. Motility, flagella, quorum sensing, and exogenous host proteins were not necessary for aggregation, but the outer membrane proteins AlpA and AlpB were. *H. pylori* lacking either *alpA*, *alpB*, or both were unable to form aggregates. While bacterial aggregation often confers tolerance to antibiotics, *H. pylori* aggregates were no more tolerant than dispersed cells to several clinically used antibiotics and human serum. Instead, we found that *alpA* mutants were highly deficient in biofilm formation, suggesting that aggregation may be a step along the *H. pylori* biofilm formation pathway.

**IMPORTANCE:**

*Helicobacter pylori* is a common human pathogen. Infection by this bacterium can lead to gastric cancers and ulcers. *H. pylori* infections present major global health challenges due to rising antibiotic resistance that complicates treatment. While bacterial aggregation is a recognized driver of antibiotic tolerance and persistence in other pathogens, its role in *H. pylori* remained unexplored. This work provides the first comprehensive characterization of *H. pylori* aggregation, demonstrating that it is a protein-mediated but flagella-independent process. We find that, unlike in many other bacteria, aggregation did not confer tolerance to tested antibiotics or serum antimicrobials, but instead may be an initial step on the pathway to forming biofilms. Characterizing *H. pylori* aggregation is a crucial step toward understanding the microbe’s life cycle and may inform novel strategies to disrupt its colonization and persistence.

## INTRODUCTION

Bacterial aggregation, the formation of a community of microbial cells that are surface-independent, is a widespread phenomenon observed in over 30 bacterial species (1). These aggregates, which can range in size from few to thousands of cells, are increasingly recognized for their frequent *in vivo* observation but uncertain role in bacterial infection processes (2). Critically, aggregation is often a precursor to biofilm formation and can confer significant survival advantages itself, including enhanced tolerance to antibiotics and host immune defenses, similar to surface-attached biofilms (3–7). Aggregation has been suggested to affect microbial ecology by concentrating effector molecules and facilitating horizontal gene transfer (8, 9). Despite its potential clinical importance, the molecular mechanisms driving aggregation and its functional consequences are not fully understood in many pathogens.

*Helicobacter pylori* is a gram-negative human pathogen that colonizes the stomach and infects about half of the human population. Infection with *H. pylori* is linked to chronic gastritis, peptic ulcer disease, gastric carcinoma, and mucosa-associated lymphoid tissue lymphoma (10). Although aggregation is a commonly observed phenomenon in *H. pylori* cultures (11), there is limited research on the topic. Evidence suggests that *H. pylori* aggregation may have a genetic basis; specific mutants, such as those lacking the chemotaxis protein CheV1 or the UDP–galactose-4-epimerase GalE, exhibit altered aggregation phenotypes (12, 13). Furthermore, aggregation can be induced exogenously by host factors, including Trefoil Factor 1 (TFF1), suggesting a potential role in host-pathogen interactions in the gastric mucosa (14). The fundamental mechanisms governing *H. pylori* aggregation are unknown, including the specific factors required, and whether it provides a survival benefit.

In this work, we set out to define the mechanism and functional significance of *H. pylori* aggregation. We developed methods to quantitatively study this process and systematically tested the roles of various bacterial proteins and structures. We report here that *H. pylori* aggregates can form non-clonally and depend on the outer membrane proteins AlpA and AlpB, but not flagella or motility. Contrary to other pathogens, *H. pylori* aggregation alone does not confer tolerance to tested antibiotics or human serum. Instead, our findings position aggregation as an essential early step in the biofilm formation pathway.

## RESULTS

### Development of a method to reliably form, quantify, and disperse *H. pylori* aggregates

**Fig 1:**
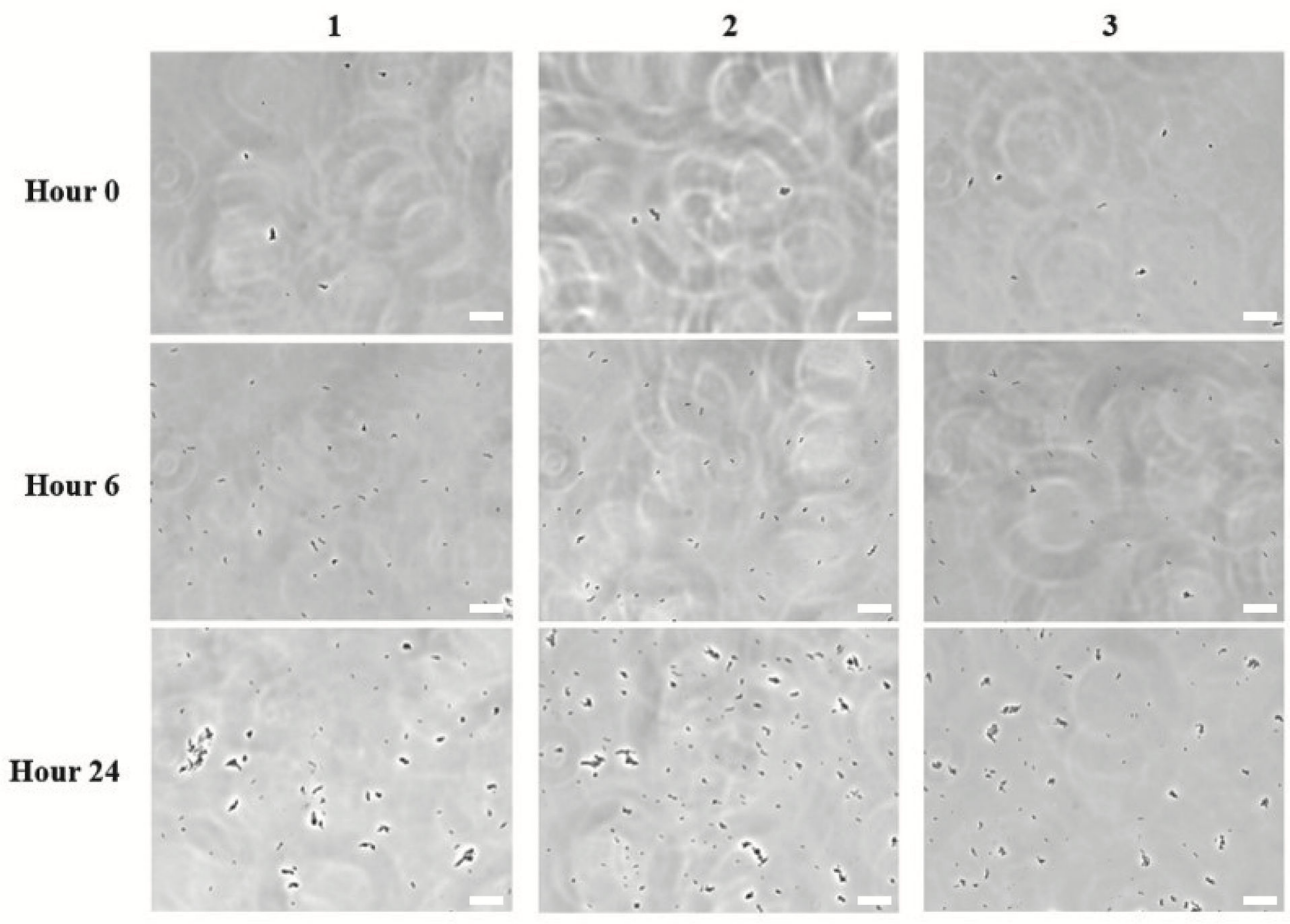
*H. pylori* forms aggregates after 24 h of growth. Three representative microscopy images of *H. pylori* G27 samples after different lengths of incubation. Samples were grown overnight for 24 h in BB10 medium with shaking, then diluted to an OD_600_ of 0.1 in fresh BB10 medium, followed by additional growth with shaking for the indicated times. Images were taken using a 40× phase objective immediately (0 h), after 6 h, or after 24 h. Images were collected from three independent biological replicates, labeled as examples 1, 2, and 3. Scale bars represent 20 µm.

While *H. pylori* aggregation *in vitro* has been frequently reported (11, 12), a standardized methodology for its reproducible analysis was lacking. We therefore sought a method to reliably form *H. pylori* aggregates. *H. pylori* strain G27 was grown overnight in liquid medium (Brucella broth [BB10]), diluted to an OD_600_ of 0.1, and then incubated with shaking for another 48 h. Immediately after diluting, small aggregates were observed that were few in number (Fig. 1). After 6 h of growth, little to no aggregation was observed, suggesting that the diluted aggregates dispersed with the addition of new medium (Fig. 1). After 24 h, *H. pylori* reliably formed large aggregates of spiral cells (Fig. 1). By 48 h of growth, cells were still aggregated, but they had turned from spiral to coccoid, an *H. pylori* morphological form that arises during starvation and stationary phase (15). We therefore used 24 h of growth after dilution to form *H. pylori* aggregates for the remainder of our studies.

**Fig 2:**
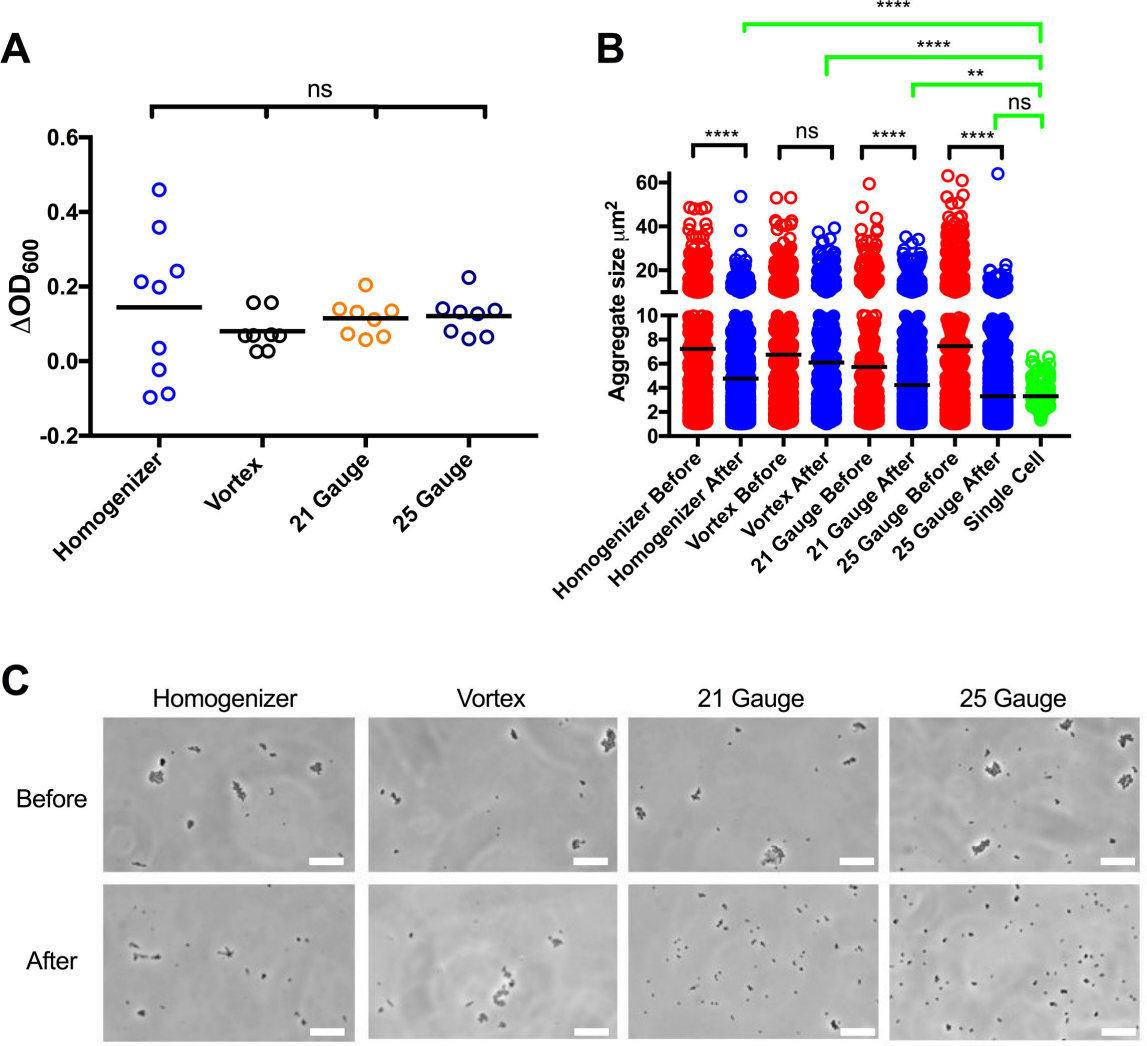
*H. pylori* aggregates are readily quantified using microscopy and dispersed using shear forces. *H. pylori* G27 aggregates were formed after 24 h of incubation and then subjected to dispersal by manual homogenization, vortexing, or shear forces via a 21- or 25-gauge needle. (**A**) Aggregation quantified by change in OD_600_ value after sedimentation, in relation to each treatment. Each open circle indicates a biological replicate. (**B**) Aggregate sizes measured via microscopy and ImageJ, before and after treatment, and compared to the size of single cells. Each identified particle in three biological replicates is represented as an open circle, with the average represented by a line. (**C**) Representative phase contrast images of samples before and after each treatment at 40× magnification. Scale bar = 20 µm. The asterisks indicate a significant difference in mean particle size according to an ordinary one-way ANOVA (**P < 0.01; ****P < 0.0001; and ns, no significant difference).

We next turned to approaches to quantitatively measure and disperse aggregates. Initially, we employed the common sedimentation assay, in which large-sized aggregated particles sink toward the bottom of the tube, resulting in a decrease in OD_600_ at the top of the liquid sample that can be quantified as ∆OD_600_ (Fig. 2A). However, this method proved insufficiently sensitive, showing little change in OD_600_ after various dispersal treatments. We therefore turned to an alternative method based on microscopy (16). For this method, particle size was measured from microscopic images analyzed by ImageJ before and after the treatment with various dispersal techniques (Fig. 2B and C). Using this quantitative assay, we tested several methods of mechanical dispersal (homogenizer, vortex, and shear force). Vigorous vortexing and homogenization failed to reduce aggregates to the size of single cells (Fig. 2B). In contrast, shear force applied to the sample by passing it through a 25-gauge needle resulted in a population that was much decreased in overall particle size and whose average size was no longer different from a control population of single cells (Fig. 2B and C). These results thus found that *H. pylori* aggregates are more sensitive to dispersal by shear force, so this approach combined with microscopy-based quantification was used going forward.

### *H. pylori* aggregates can form non-clonally and require surface-exposed proteins

**Fig 3:**
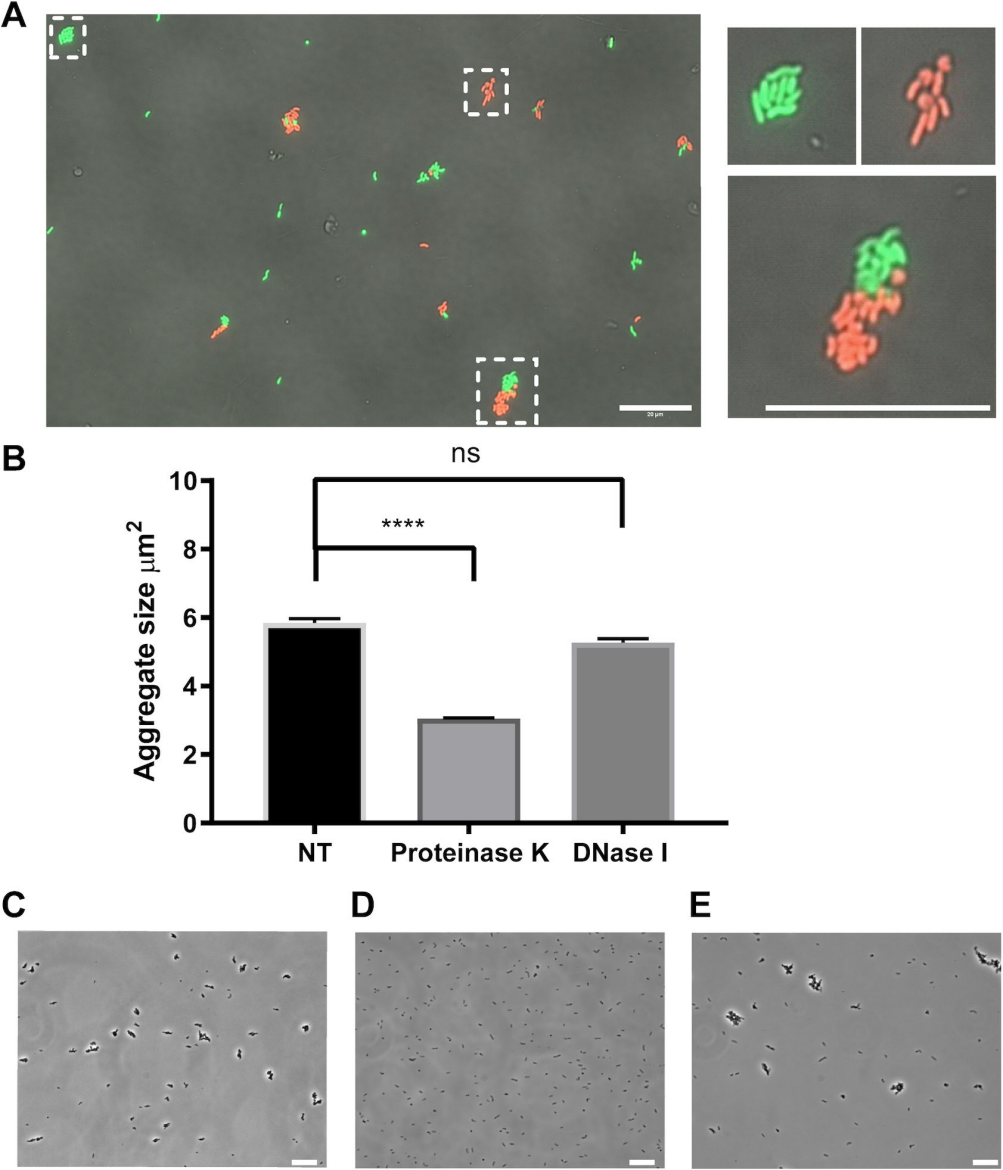
*H. pylori* aggregates can form non-clonally and depend on proteins. (**A**) *H. pylori* strains expressing either GFP or RFP were mixed and allowed to form aggregates. Images were collected using confocal microscopy at 40× magnification. The marked area shows magnified views of aggregates containing only red, only green, or both red and green *H. pylori*. Images are representative of three biological replicates. (**B**) *H. pylori* G27 was grown overnight, diluted to an OD_600_ of 0.1 in fresh medium, and either 0.1 mg/mL of Proteinase K, 1.0 mg/mL of DNase I, or nothing (NT) was added for the 24-h incubation growth period. Aggregate size was quantified by microscopy and ImageJ. Data are the mean of three biological replicates, with at least 3,000 particles each. Error bars represent the standard error of the mean. Statistical analysis was calculated according to a Mann-Whitney test (****P < 0.0001; ns, no significant difference). (**C–E**). Representative images of *H. pylori* aggregates after no treatment (**C**), Proteinase K (**D**), or DNase I (**E**) at 40× magnification. Scale bar indicates 20 µm.

To explore whether *H. pylori* aggregates form from clonal expansion of a single cell or from the adhesion of multiple cells, we co-cultured isogenic strains of *H. pylori* expressing either GFP or RFP. Analysis of the resulting aggregates by fluorescent microscopy revealed a mixed population; some aggregates were solely comprised of GFP-positive or RFP-positive cells, while others contained both (Fig. 3A). The presence of these mixed aggregates suggests that aggregates can form from the union of distinct cells. While this finding does not eliminate the possibility of aggregation arising from clonal growth, it establishes that clonal growth is not the sole mechanism for aggregate formation.

We next sought to determine the molecular components critical for aggregate formation. Based on established protocols for studying *H. pylori* biofilms (17), we treated cultures with Proteinase K or DNase I. Previous work has shown that *H. pylori* biofilm formation is not affected by DNase I, but is dependent on surface proteins, which can be degraded by the serine proteinase Proteinase K at doses that only impact surface proteins (17, 18). To evaluate how these treatments affected *H. pylori* cell aggregates, these treatments were added at the start of the 24-h incubation. Proteinase K completely abolished aggregation, while DNase I treatment had no significant effect on aggregation (Fig. 3B and C). Notably, addition of these enzymes to pre-formed aggregates did not fully disperse them (data not shown). These results establish that initial aggregate formation is dependent on surface proteins but not on eDNA.

### Neither flagella nor motility is necessary for *H. pylori* aggregation

**Fig 4:**
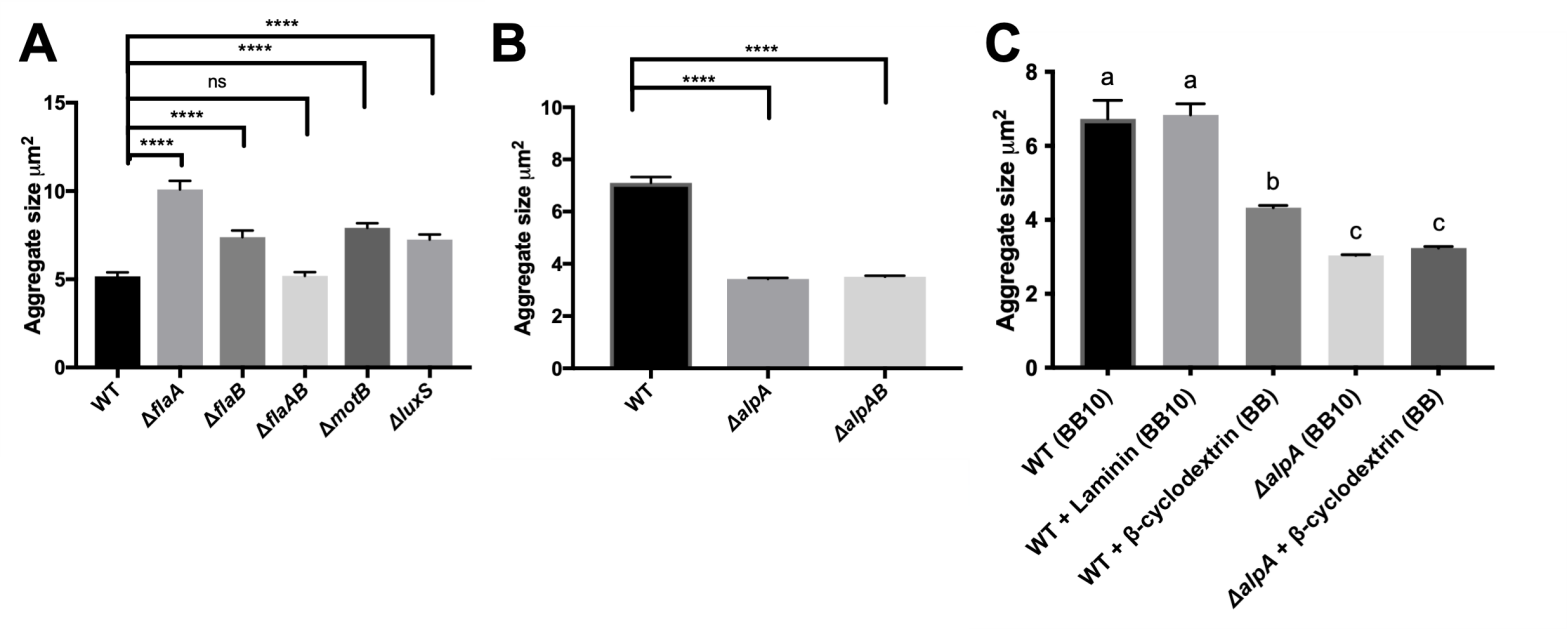
*H. pylori* aggregate formation requires *alpAB* but not genes for flagella or motility, or exogenous serum components. (**A and B**) Aggregate formation by *H. pylori* G27 WT or isogenic mutants after 24 h of incubation. Mean aggregate size of three independent biological replicates was quantified using microscopy and ImageJ, using at least 1,000 particles for each strain or medium. Error bars show standard error of the mean. Statistical analysis used in panel **A**, an ordinary one-way ANOVA with comparison to the WT control (****P < 0.0001; ns, no significant difference); in panel **B**, an unpaired *t*-test (****P < 0.0001). (**C**) *H. pylori* G27 WT and Δ*alpA* were cultured overnight in different conditions (BB10, BB10 + 10 µL/mL Laminin, or BB + 0.333 mg/mL β-cyclodextrin), diluted to OD_600_ of 0.1 in matched fresh medium, and left to grow for 24 h. Over 2,800 particles were analyzed for each group. Statistical analysis was performed using an ordinary one-way ANOVA with multiple sample comparison using Tukey’s correction. Different letters indicate different statistical groups.

In many bacteria, surface structures like flagella play a direct role in cell adhesion and aggregation. While flagella act as structural scaffolds in *H. pylori* biofilms (17, 19), their role in aggregation is unknown. We therefore analyzed aggregate formation by a panel of motility mutants. *H. pylori* flagella are composed of two flagellins, FlaA and FlaB (20, 21). A mutant lacking both flagellins (Δ*flaAB*) has no flagella; this mutant formed aggregates at wild-type (WT) levels and sizes (Fig. 4A), demonstrating that flagella are not required for aggregation. *motB* encodes for stator units of the flagella motor, and its deletion leads to a flagellated but non-motile phenotype (22). *motB* mutants also did not have a defect in aggregation but instead had slightly but significantly elevated aggregation (Fig. 4A). Indeed, mutants with partial or non-functional flagellar systems (Δ*flaA*, Δ*flaB*, or Δ*motB*) all exhibited significantly increased aggregation (Fig. 4A). Other work has shown that *H. pylori* uses quorum sensing to disperse biofilms, for example, strains lacking *luxS*, which are unable to synthesize quorum sensing molecule AI-2 (23), form elevated biofilms (24). We therefore tested an *H. pylori* Δ*luxS* strain and found it aggregated to a level that was modestly but significantly higher than WT (Fig. 4A). Overall, these results suggest that flagella and motility are not needed for aggregate formation but instead, like quorum sensing, may play a role in limiting aggregation.

### The outer membrane proteins AlpA or AlpB are necessary for aggregation

Having ruled out a role for flagella in *H. pylori* aggregate formation, we employed a candidate approach to identify factors essential for this process, testing various mutants lacking genes for outer membrane proteins, chemotaxis signaling, glycosylation, and flagella function including *alpA*, *alpB*, *hopQ*, *sabA*, *babA*, *pseE*, *flgK*, and *cheV123*. This analysis revealed that mutants lacking the genes for the outer membrane proteins AlpA or both AlpA and AlpB (referred to as AlpAB) were severely deficient in aggregation, with cultures consisting almost entirely of single cells, while other strains behaved as wild type (Fig. 4B). AlpA and AlpB (adherence-associated lipoproteins A and B) are outer membrane proteins that share over 46% identity in their amino acid sequences with each other (25). AlpA and AlpB are required for effective colonization in guinea pigs (26) and mice (27). Their functions in adhesion have been shown in several studies, including adherence to human gastric tissue (25) and binding to laminin, a glycoprotein in the mammalian basement membrane that is also present in human and bovine serum (28–30), but had not been noted for aggregate formation.

Because fetal bovine serum (FBS) is used regularly for *H. pylori* growth and in these aggregation studies, we considered that serum proteins, specifically laminin present in FBS, might bind to AlpAB during aggregation and act as a bridge between cells. To test the role of laminin in aggregate formation, high concentrations of mouse laminin-1, a known binding partner of AlpA and AlpB (28), were added during aggregate formation. We hypothesized that a high concentration of laminin would saturate the AlpA and AlpB binding sites, reducing the chance of different *H. pylori* bacteria binding to the same laminin molecule. WT *H. pylori*, however, was able to aggregate equally with and without laminin (Fig. 4C). We then explored whether *H. pylori* cells would aggregate when grown in medium that lacked FBS. FBS contains growth factors that are required for robust *H. pylori* growth, but the microbe is able to grow to a modest level when medium instead contains β-cyclodextrin (31). As reported previously, *H. pylori* growth was not as robust in serum-free medium (BB) as in medium containing it. *H. pylori* was still capable of aggregating, however, albeit to levels that were not as high as in medium with serum (Fig. 4C). Furthermore, loss of *alpA* significantly decreased aggregation size as compared to WT in either medium (Fig. 4C). Taken together, these results support that aggregation is not dependent on binding exogenous proteins like laminin and appears instead to be an intrinsic property of *H. pylori*.

**Fig 5:**
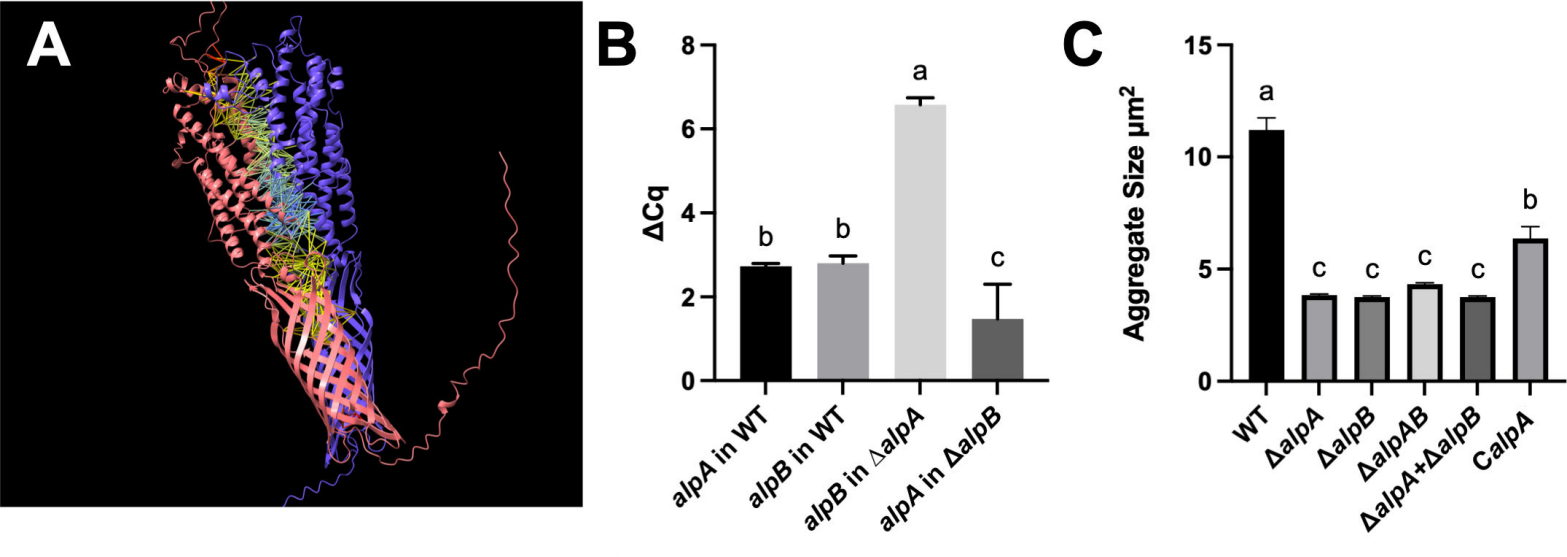
AlpA and AlpB are predicted to interact, but cross-cell interactions may not underlie aggregation. (**A**) Predicted structures of AlpA (purple) and AlpB (red) heterodimer. Lines at the interface indicate predicted interactions, with blue demarking higher confidence predictions. (**B**) Expression of *alpA* or *alpB* was evaluated using qRT-PCR and compared between WT, Δ*alpA,* or Δ*alpB* mutant backgrounds. ΔCq values were calculated using the 16S gene for normalization compared to the gene indicated in parentheses. Error bars indicate standard deviation. One biological replicate with six samples is shown, representative of three biological replicates. (**C**) Quantification of aggregation in samples of *H. pylori* Δ*alpA*, Δ*alpB*, Δ*alpAB*, or the Δ*alpA* mutant complement (*CalpA*) cultured individually or Δ*alpA* co-cultured with Δ*alpB* (Δ*alpA* + Δ*alpB*). Error bars represent standard error of the mean. Over 3,000 particles were analyzed for each group. For both panels **B and C**, statistical analysis was performed using a one-way ANOVA with Tukey’s multiple sample correction (GraphPad Prism). Letters above the bars indicate statistical groups, for example, a is different from b.

Because AlpA and AlpB can form a complex (32), we postulated that AlpA and AlpB could facilitate direct cell-cell interactions, either on the same or different cells, as Senkovich et al. have also suggested (28). *In silico* structural analysis using ColabFold predicted that AlpA and AlpB could form stable heterodimers (Fig. 5A), lending some support for this hypothesis. To explore this idea experimentally, we created single-gene deletion mutants lacking either *alpA* or *alpB* and evaluated how loss of one affected transcription of the other via qRT-PCR (Fig. 5B). *alpA* and *alpB* were both expressed to similar levels in WT, with a Cq of ~30 compared to 28 for 16S, and thus a ∆Cq of 2 (Fig. 5B). Deletion/insertion of *alpB* led to a modest increase in *alpA* expression: the ∆Cq decreased to 1, suggesting the expression increased by twofold (Fig. 5B). *alpB*, in contrast, decreased dramatically in expression, with a ∆Cq of ~6, although the gene was still expressed (Cq = ~32). While it is unknown how these changes in transcription relate to protein expression, these results suggest that the ∆*alpA* mutant may express less *alpB* than WT, but the ∆*alpB* mutant expresses near-normal amounts of *alpA*. Each single mutant was then evaluated for aggregation and found to be deficient to the same level as each other or deletion of both (Fig. 5C). Complementation of *alpA* at an intergenic locus allowed significant restoration of the ∆*alpA* mutant aggregation, indicating loss of this gene was at least partially responsible for the aggregation defect. We then tested whether there was evidence that AlpA on one cell might bind to AlpB on another, by co-culturing the two mutants and evaluating for restoration of some aggregation. The co-cultured samples, however, did not aggregate to a greater extent than the single mutants (Fig. 5C). This result suggests that while AlpA and AlpB are both essential, we did not find evidence that they interact to bridge adjacent cells.

### Aggregates do not confer protection against antimicrobials

**Fig 6:**
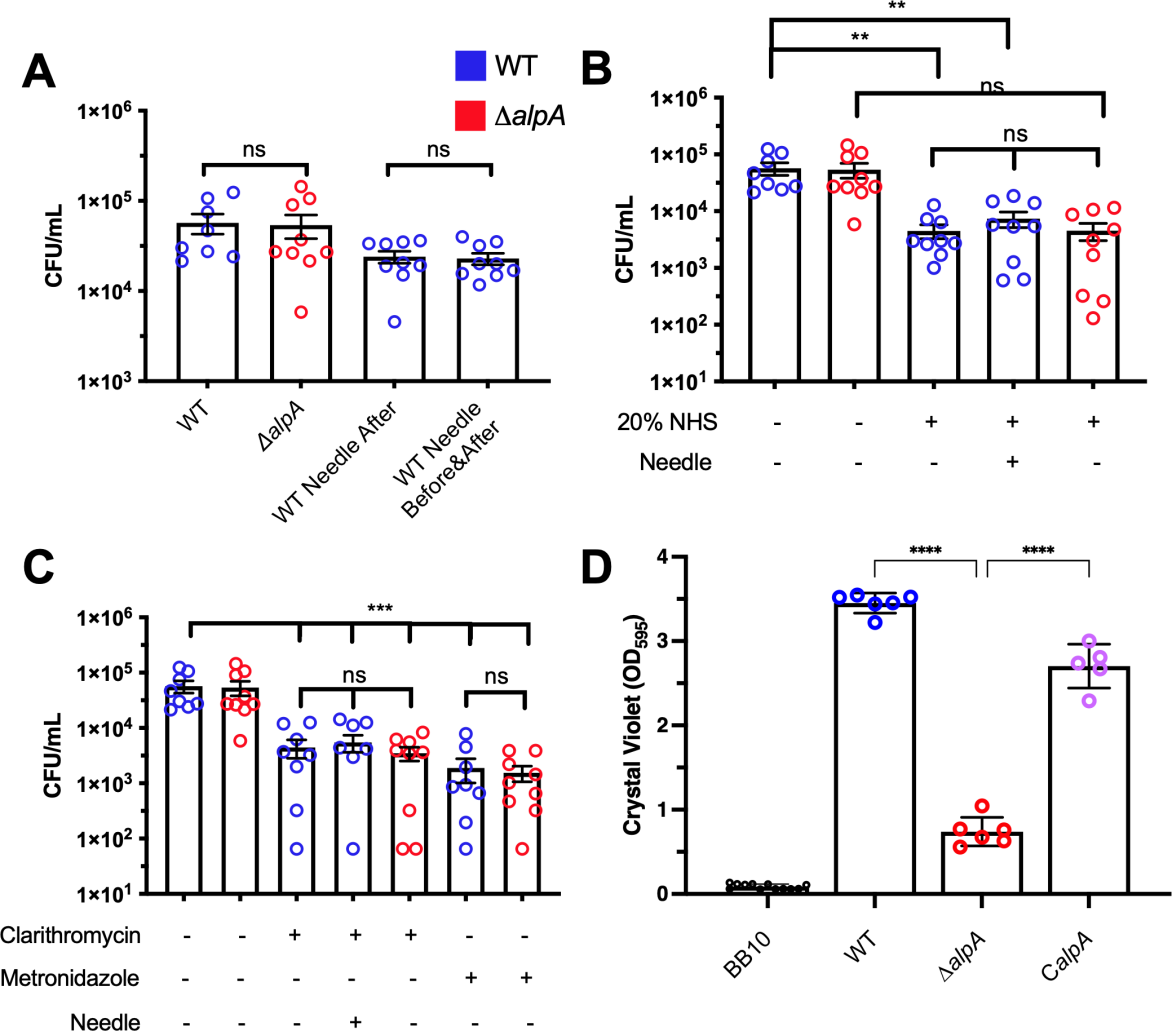
Aggregation plays a role in biofilm formation but does not increase survival against human serum or antibiotics. (**A–C**) *H. pylori* G27 WT (blue) or Δ*alpA* (red) samples were grown overnight to form aggregates, confirmed by microscopy, and diluted to an OD_600_ value of 0.1 in fresh BB10. Samples were either left aggregated or dispersed with a needle and then treated with the indicated challenges for 1.5–2 h. (**A**) *H. pylori* WT or Δ*alpA* samples at the same OD_600_ were plated to determine colony-forming units (CFU) with and without needle dispersion before the 1.5- to 2-h incubation period. (**B and C**) *H. pylori* WT or Δ*alpA* were left untreated or dispersed by shear force (needle) and then treated with active normal human serum (NHS) in panel **B** or clarithromycin or metronidazole in panel **C**, followed by dilution and plating for CFU. Error bars show standard error of the mean. Statistical analysis is shown according to a one-way ANOVA (**P < 0.01; ***P < 0.001; and ns, no significant difference). (**D**) *H. pylori* G27 WT (blue), Δ*alpA* (red), and an Δ*alpA* mutant complemented with *alpA* at the intergenic locus (*CalpA*, purple) were grown in a 96-well plate for 3 days. Wells were washed with PBS and adhered biomass was stained with crystal violet. Data are presented as a representation of three or more biological replicates, with five technical replicates each. The negative control is BB10 medium alone. Indicated comparisons to the Δ*alpA* strain were done via an unpaired Student’s *t*-test, ****P < 0.001. The ROUT outlier test was used to remove outliers at *Q* = 5%. Error bars represent standard deviation.

A well-documented boon of bacterial aggregation is enhanced tolerance to antimicrobial insults (3–6). We therefore investigated whether *H. pylori* aggregation conferred survival advantages against components of the innate immune system and antibiotics by comparing the response to such insults between WT aggregates, shear-forced dispersed WT aggregates, and the non-aggregating *alpA* mutant. Control experiments established that WT and the *alpA* mutant resulted in the same number of CFU per OD_600_ unit, suggesting these two samples could be readily compared and that treatment with the needle during shear force resulted in a ~0.5 log decrease of CFU numbers (Fig. 6A). CFU numbers decreased by similar amounts regardless of whether the needle treatment was applied after the 1.5- to 2-h incubation period or both before and after (Fig. 6A). With these control values in hand, we then challenged aggregated and single cell *H. pylori* samples with several agents. First, normal human serum (NHS) was used, as it contains antimicrobial peptides and complement components, conditions that *H. pylori* experiences *in vivo* when colonizing the stomach (33). Treatment with NHS resulted in a ~10-fold decrease in *H. pylori* numbers compared to untreated, but there was no difference in survival between WT, dispersed WT, or Δ*alpA* (Fig. 6B). These data suggest that aggregation does not increase survival against NHS. Next, we performed a similar experiment by testing survival against two antibiotics commonly prescribed as treatments for *H. pylori* infection: the macrolide clarithromycin and the nitroimidazole metronidazole. Clarithromycin binds the 23S rRNA, thereby halting protein translation (34). Metronidazole leads to cytotoxic production that destabilizes DNA (35). Aggregates were developed for 24 h, left intact or dispersed, and then treated for 2 h with clarithromycin or metronidazole at amounts that were 60× or 9× the minimum inhibitory concentrations, respectively (36). As with the serum, we observed no difference in survival in aggregated or dispersed WT or Δ*alpA* against clarithromycin or metronidazole (Fig. 6C). Taken together, these results suggest that *H. pylori* aggregates formed under these conditions are not substantially tolerant to the external stresses tested.

### *H. pylori* Δ*alpA* forms less biofilm than WT

Given that aggregation did not confer an obvious advantage to external insults, we hypothesized that it might instead act as a precursor to surface-attached biofilm formation, a known strategy for persistence in other bacteria as seen in *Pseudomonas aeruginosa* (7) and *Sinorhizobium meliloti* (37). A biofilm assay was performed on WT, the aggregation-deficient *alpA* mutant, and a complemented *alpA* mutant strain generated by placing a copy of *alpA* under its own promoter at a neutral intergenic locus. Biofilms were formed over a 3-day period in a 96-well plate, followed by quantification via crystal violet staining of adherent bacteria. A fourfold decrease in crystal violet staining was observed in the Δ*alpA* strain compared to WT, a defect that was partially recovered in the complemented strain (Fig. 6D). Of note, *alpA* mutants did not display any general growth defects, for example, in the planktonic state. This significant deficiency is consistent with the idea that AlpA is essential for cell-cell aggregation in liquid culture for the formation of mature surface-attached biofilms, supporting the idea that *H. pylori* aggregates form as part of the biofilm pathway.

## DISCUSSION

While bacterial aggregation has been extensively studied in model bacteria such as *P. aeruginosa,* its role in the gastric pathogen *H. pylori* was poorly understood. In this study, we fill this gap by developing methods to quantitatively study aggregation in *H. pylori*. Our work reveals that *H. pylori* aggregation is a protein-dependent process mediated by the highly conserved outer membrane adhesins AlpA and AlpB, and rather than serving as a protective strategy against the antimicrobials, it appears to function as a critical precursor to biofilm development.

We established that aggregates are formed when *H. pylori* strain G27 grows for 24 h in BB10 medium (Fig. 1A). This growth amount places the bacteria in late log or early stationary phase (38) when large aggregates are formed, consistent with observations in other bacteria (6). A microscopy-based quantification method proved sensitive for measuring aggregate disruption, more so than traditional sedimentation assays (Fig. 2A). The lack of sensitivity of the sedimentation assays might be because most *H. pylori* aggregates are relatively small at ~5–10 µm^2^, compared to single cells at 3 µm^2^, and so may not sediment much differently. Finally, we found that shear force can disperse *H. pylori* aggregates while homogenization or vortexing were not able to cause significant dispersal (Fig. 2B and C). This finding supports the idea that *H. pylori* cells in aggregates are physically interacting and not just near each other.

Our experiments demonstrated that *H. pylori* aggregation depended on surface proteins, as evidenced by complete inhibition with Proteinase K treatment during aggregate formation (Fig. 3B). This protein-dependent mechanism parallels findings in the closely related pathogen *Campylobacter jejuni* (39) but contrasts with aggregation mechanisms in other species (*B. subtilis*, *S. epidermidis*, and *N. meningitidis*) that utilize eDNA as a primary structural component (40–42). This finding does not preclude the possibility that eDNA, polysaccharides, or other proteins buttress the mature aggregate.

Our work here shows that flagella are dispensable for *H. pylori* aggregation (Fig. 4A), in contrast to their important role in the *H. pylori* biofilm structure (17). This finding may suggest that flagella’s physical role may be more important in later stages of the aggregate-biofilm process. It also supports the idea that the proteins required for aggregation are not found on the flagella. This finding differs from that in *C. jejuni*, which aggregates via glycosylated flagellins (43). The differences between *C. jejuni* and *H. pylori* may be due to differences in the flagellar structure. Specifically, *H. pylori* flagellins are surrounded by a membrane sheath, which may block direct interactions between flagellins (44). Indeed, motility-deficient mutants (Δ*flaA*, Δ*flaB*, and Δ*motB*) and a quorum-sensing mutant (Δ*luxS*) had no aggregation defects and actually exhibited enhanced aggregation. Genes for flagella-related proteins have been found to be differentially regulated in biofilm and adherence work. Previous transcriptomic analysis of the *H. pylori* biofilm cells compared to planktonic ones found upregulation of the gene for one flagellin, *flaB,* but not the other (*flaA*) (17). *flaB* in *H. pylori* was downregulated in aggregates induced via TFF1 compared to cells not incubated with TFF1 (14). Other genes that regulate FlaA were also differentially expressed. Specifically, *flgM*, which encodes an anti-sigma factor for *fliA*, was also overexpressed in biofilms, suggesting that, like in aggregates, larger communities form when FlaA is lessened (17). This effect contrasts with the role of *motB* in the *H. pylori* biofilm, in which mutants lacking *motB* and flagellar formation genes display poor biofilm formation (45) but aggregate similarly or better than WT (Fig. 4A). These findings reveal a fundamental distinction between the structural requirements for aggregation vs biofilm formation in *H. pylori*.

The enhanced aggregation observed in flagellar mutants (Δ*flaA*, Δ*flaB*, and Δ*motB*) presents an intriguing parallel to phenomena in other bacterial species. In *Pseudomonas aeruginosa* and *Vibrio cholerae*, flagellar mutations increase exopolysaccharide production, enhancing community formation (46, 47). However, investigating whether this mechanism occurs in *H. pylori* is complicated by differences in matrix composition. Proteins appear to be the primary structural component of the *H. pylori* biofilm (17, 18) and from work here, the matrix that holds together aggregates. Specifically, our analysis of aggregates via proteinase K and DNase I (Fig. 3) demonstrates that surface proteins are essential for aggregate formation, while DNase I had no effect (Fig. 3), supporting a protein-centric model. This finding does not rule out a supporting role for eDNA, polysaccharides, or additional proteins in mature aggregate architecture. Indeed, the matrix composition of *H. pylori* communities remains incompletely characterized. The increased aggregation in flagellar mutants could result from increased cell proximity and/or duration of contact, facilitating cell-cell interactions. This interpretation aligns with observations that hypermotile mutants (Δ*pilO*) aggregate less, while mutants with chemotaxis defects (Δ*cheV1*) aggregate more (12, 48). Upregulation of adhesins, activation of motility-dependent signaling pathways, or changes in outer membrane properties may also play a role. Future transcriptomic or proteomic comparisons between WT and flagellar mutants during aggregate formation could reveal whether motility loss triggers compensatory upregulation of other factors, similar to regulatory connections observed in other species.

We found that both the AlpA and AlpB outer membrane adhesins are critical for aggregation and biofilm formation (Fig. 4B). This conclusion was reached using independent *alpA*, *alpB*, and *alpAB* mutants as well as genetic complementation. Other groups have reported similar findings with Δ*alpA* and Δ*alpB* mutants in strain 26695m and clinical isolate TK1402 (28, 49). We noted that loss of *alpA* did not confer a general growth defect, but our work did not evaluate whether the lowered crystal violet staining was due to decreased numbers of cells in the biofilm or less matrix, and analysis that can lend insights into defects as reported recently (50). AlpA and AlpB are highly conserved throughout *H. pylori*, present in 100% of 200 clinical isolates, whereas eight other OMPs ranged in frequency from 35% to 73% (51). *alpAB* are predicted to be in a two-gene operon, with about 2,400 base pairs to the next gene oriented in the same direction, *hopG-2*. Our results with qRT-PCR suggest that changes to *alpA* transcription, for example, by replacing the gene with an antibiotic resistance cassette, can affect the likely downstream *alpB* (Fig. 5B). Although AlpA and AlpB are reported to bind laminin, this function does not seem to be key during aggregate formation, based on studies here that laminin addition did not change aggregate formation (Fig. 4). Indeed, aggregation can happen without any FBS proteins, strongly suggesting that aggregation is a *H. pylori*-intrinsic property (Fig. 4C). We did note that aggregate size did decrease modestly when formed in the absence of FBS, but this difference may result from less bacterial growth rather than any specific roles for FBS proteins. AlpA and AlpB thus contribute to the list of genetic factors required for *H. pylori* aggregation, in addition to GalE and CheV1 (12, 13). Teasing apart the roles of these proteins will require further experiments. Aggregation observed in *H. pylori* appears to be distinct from what Secor et al. describe as depletion aggregation (5). In depletion aggregation, cells in polymer-rich solutions can be held together not only by intercellular interactions but also by the system maximizing entropy by creating the most room for polymers as well. The forces of the polymers surrounding cells that have come together hold them there. Here, we show that *H. pylori* cells can hold themselves together without exogenous factors, supporting other findings that aggregation has a cell-intrinsic genetic basis.

As has been suggested by other groups (28), our modeling also suggested that AlpA and AlpB could possibly interact (Fig. 5A). To test this idea, we first verified that our *alpA* and *alpB* single mutant strains were still expressing the other via qRT-PCR. We note that *alpB* was expressed at lower levels in the Δ*alpA* background, while *alpA* in the Δ*alpB* background increased transcriptional levels (Fig. 5B), which may explain why complementing Δ*alpA* did not completely rescue aggregation to WT levels (Fig. 5C). Additionally, our co-culture experiments failed to demonstrate *trans* complementation between Δ*alpA* and Δ*alpB* mutant strains (Fig. 5C). This finding suggests that a cell must express both proteins for aggregate formation. The recent discovery that AlpAB proteins are glycosylated during outer membrane transport suggests another layer of regulation that may influence their aggregation function in adhesin-binding abilities (52). However, it is not known whether *alpA* and *alpB* are transcriptionally regulated. We were not able to find significant *alpA*/*alpB* upregulation or downregulation in several RNA-Seq data sets, including biofilm ones (17, 19), hinting that increased expression may not be required for aggregation or biofilm formation. Overall, we show that AlpA and AlpB are important for aggregation, but more work will need to be done to determine how these proteins facilitate aggregation and whether there is any regulation on these proteins.

One vexing problem is squaring away *H. pylori’s* propensity to aggregate with its reliance on motility in infection. The glycoprotein TFF1, commonly found in the human stomach, binds specifically to the *H. pylori* rough lipopolysaccharide (53) and induces aggregation *in vitro*. This property of TFF1 may function as a defense system similar to the human lectin-like protein Zg16, which induces bacterial aggregation, thereby hindering bacterial penetration of the colon epithelium (54). AlpA/AlpB mutants are deficient in adherence to a gastric biopsy segment (13), so a host factor that mimicked the structure of the AlpA/B host-cell receptor may induce aggregation in a manner similar to TFF1, although there is no direct evidence for this idea. Because of this seemingly downside of aggregation, we hypothesized that this phenomenon improved cell survival against insults as a tradeoff, as seen in many other bacteria. We were unable, however, to detect a protection function for aggregation against exogenous threats, including serum components, clarithromycin, and metronidazole (Fig. 6B and C). This contrasts with not only the *H. pylori* biofilm, which increases survivability, but also with aggregation in *P. aeruginosa* and *Staphylococcus aureus* (5, 6, 55, 56). Other possible functions for aggregation may be to confer protection from other types of insults, for example, phagocytosis as seen in *P. aeruginosa* and *S. aureus* (57), or perhaps it increases the concentration of extracellular proteins. Urease, an enzyme *H. pylori* uses to increase the pH of the highly acidic stomach, might be more effective for a group of cells than single cells. Finally, a third idea discussed above is that aggregation is a step along the pathway of biofilm formation, but only mature biofilm cells are protected from antibacterial insults. Our findings have provided insight into *H. pylori* aggregate formation as well as ways to manipulate aggregates and thus will pave the way to understanding community behavior in this important gastric pathogen.

## MATERIALS AND METHODS

### General *H. pylori* growth

All strains used in this work are described in Table 1. Strains were cultured on Columbia Horse Blood Agar (CHBA) (Difco) plates with 50 μg/mL cycloheximide, 10 μg/mL vancomycin, 5 μg/mL cefsulodin, 2.5 units/mL polymyxin B, 0.2% (weight/volume) β-cyclodextrin, and 5% defibrinated horse blood (HemoStat) or were cultured in liquid growth medium consisting of 90% Brucella broth (BB) and 10% heat-inactivated Fetal Bovine Serum (BB10). *H. pylori* was cultured at 37°C under microaerobic conditions of 5% O_2_, 10% CO_2_, and 85% N_2_. When needed, antibiotics were used at final concentrations of 15 µg/mL for kanamycin, 25 µg/mL for erythromycin, or 13 µg/mL for chloramphenicol. Unless stated otherwise, liquid cultures were grown shaking at 200 rpm.

**TABLE 1:** Bacterial strains used in this work^*a*^

*H. pylori* strain	KO collection number	Genotype or description	References and/or sources
G27		Wild type	(58)/from Nina Salama
G27-GFP	KO531	G27 pTM115-GFP	(59)
G27-RFP	KO1562	G27 pTM115-RFP	(60)
Δ*motB*	KO489	G27 Δ*motB*::*kan-sacB*	(22)
Δ*flaA*	KO1984	G27 Δ*flaA*::*cat*	This work
Δ*flaB*	KO1985	G27 Δ*flaB*::*cat*	This work
Δ*flaAB*	KO1988	G27 Δ*flaB*::*cat* ∆*flaA*::*aphA3*	This work
Δ*luxS*	KO1791	G27 Δ*luxS*::*erm*	This work; SS1 allele from reference 61
Δ*alpA*	KO2018	G27 Δ*alpA*::*aphA3*	This work
Δ*alpAB*	KO2019	G27 Δ*alpAB*::*erm*	This work
Δ*alpB*	KO2057	G27 Δ*alpB*::*cat*	This work
C*alpA* IG::*alpA*	KO2150	KO2018 with intergenic *alpA-erm*	This work

^*a*^ *H. pylori* strains used in this work.

### Aggregate formation

For aggregate formation, *H. pylori* was cultured overnight in BB10 with shaking under microaerobic conditions. OD_600_ was determined, and cultures were diluted to an OD_600_ of 0.1 in fresh BB10 unless otherwise indicated, using 3 mL BB10 in a 15 mL Falcon tube. Cultures were incubated with shaking for up to 48 h. For experiments after the initial time course of aggregation, 24 h of aggregate formation was used.

For dual-strain aggregate formation, *H. pylori* G27 with pTM115-based plasmids expressing either GFP (59) or RFP (60) were grown overnight separately in BB10 with shaking. Cultures were then diluted to an OD_600_ of 0.1 in fresh BB10 and combined in equal amounts in the same tube. The culture was incubated for 24 h with shaking, then imaged with a Zeiss AxioImager Z2 at 40× magnification. Images were collected with a Zeiss Axiocam 506.

For aggregate formation with laminin, *H. pylori* cells were grown overnight in BB10, then diluted to an OD_600_ of 0.1 in 3 mL BB10 in a 15 mL Falcon tube. Thirty micrograms of Corning Ultrapure Mouse Laminin (CB40239) were added to each sample, followed by incubation with shaking under *H. pylori* culture conditions for 24 h.

*H. pylori* cells were collected from a CHBA plate and inoculated directly into 3 mL BB supplemented with 10 μL of 100 mg/mL β-cyclodextrin (TCI) dissolved in DMSO, as used previously to promote growth in the absence of serum (31). After 24 h of growth with shaking, samples were diluted to an OD_600_ of 0.1 at 3 mL in fresh BB + β-cyclodextrin medium and incubated with shaking for another 24 h of growth.

### Aggregation measure using sedimentation

After culturing to form aggregates as described above for 24 h, the optical density at the top of the sample was measured by removing 90 µL from the top of the tube. The samples were then left to stand statically in an upright position in a microaerobic incubator for 3 h to allow aggregate settling. After this period, 90 µL from the top of the tube was again collected for OD_600_ measurement, and the difference between the two measurements was calculated as ∆OD.

### Aggregation measurement using microscopy

Two microliters of *H. pylori* sample were imaged using phase contrast with a Nikon Eclipse E600 with 40× magnification. Images were collected with a Hamamatsu C7472-95 digital camera using μManager software (62). Ten images were taken per sample, chosen in a random grid pattern. Binning was set to 1 × 1, with less than 50 ms of exposure time. Aggregation was quantified by measuring the size of particles in a sample using ImageJ (63). The scale was set to 5.333 pixels/μm. The ImageJ threshold function was applied to each image, which was then manually corrected by either erasing particles deemed not to be *H. pylori* or by drawing a bridge between particles that were obviously connected. The analyze particle tool was used, with the lower size cutoff set to 1 μm^2^ and no upper limit. Control samples were generated by analyzing the area measurements of 200 G27 WT single cells.

### Aggregate disruption

For disruption with the manual homogenizer, 1 mL of culture was placed into a 7 mL Pyrex glass homogenizer. Moderate force was applied to 100 rotations. For disruption with the vortex mixer, 0.5 mL of culture in a 1.5 mL Eppendorf tube was vortexed with a VX100 Labnet at max speed for 5 min. For disruption with the needle, 0.5 mL of culture was forced through either a 21- or 25-gauge BD PrecisionGlide needle 15 times with a BD 1 mL Slip Tip syringe.

### Enzymatic treatment

*H. pylori* cells were grown overnight in BB10, then diluted to an OD_600_ of 0.1 in 3 mL BB10 in a 15 mL Falcon tube. Either DNase I lyophilized powder (Sigma-Aldrich) or Proteinase K lyophilized powder (Sigma-Aldrich) was added directly to the 3 mL sample to achieve a concentration of 1.0 or 0.1 mg/mL, respectively. Samples were then briefly vortexed and incubated for 24 h.

### Strain construction

Mutant strains were generated in *H. pylori* G27 via natural transformation and homologous recombination, as previously described (64). All primers used for mutant construction are listed in Table 2.

**TABLE 2:** Primers used in this work

Name	Sequence	Purpose
XL124	5′cgggatccctcatgaaaaacaatcctaggatagctctcca	Creation of Δ*alpA* mutant
XL125	5′CTATCCACTATATCATAAGAAGTTAAAGCGGCGATATTGGT	
XL126	5′TCGCCGCTTTAACTTCTTATGATATAGTGGATAGATTTATGATATAATGAGTTATCAAC	
XL127	5′GCAAAACTCCCTCCGCAGGACGCACTACT	
XL128	5′GTCCTGCGGAGGGAGTTTTGCTATGGCGCA	
XL129	5′acgcgtcgactcaaacaatgaagcgatcatgttgtaattgc	
XL294	gctatcggtggggttttagg	Amplifying *luxS* allele
XL299	ctctaaaaaatctttactaaaattgaactcgtg	
XL461	5′GTTGCGAGGGCCATTAAA	Creation of Δ*alpA* mutant
XL462	5′CATAGTATCGGCTAGCGCAAAGGGC	
XL463	5′TTGCGCTAGCCGATACTATGTTATACGCCAACT	
XL464	5′CGGAAATACTTCACCTAAAACAATTCATCCAGTAAAATATAATATTTTATTTTCT	
XL465	5′TGAATTGTTTTAGGTGAAGTATTTCCGTCCTTATAGC	
XL466	5′ATTCTTGCTGATGTTTTGACTCAAA	
XL468	5′TCCTTATAAAATGCTAGCGCAAAGGGC	Creation Δ*alpAB* mutant
XL469	5′CTTTGCGCTAGCATTTTATAAGGAGGAAAAAATAAAGAGGG	
XL470	5′AGGGCGGAAGTAAAACAAGTTAAGGATGCAGTTTATG	
XL471	5′TTAACTTGTTTTACTTCCGCCCTTATAGCG	
XL472	5′ATTAGCGGCATTCACCCC	
XL294	5′GCTATCGGTGGGGTTTTAGG	Creation Δ*luxS* mutant
XL299	5′CTCTAAAAAATCTTTACTAAAATTGAACTCGTG	
pUC19fwd	5′GTCATAGCTGTTTCCTGTGTG	Creation Δ*alpB* mutant
pUC19rev	5′AGGAGCGGCTACTGGCCGTCGTTTTACAAC	
AlpBUPfwd	5′GACGGCCAGTAGCCGCTCCTATCAAAAC	
AlpBUPrev	5′CTATATCATAAAAGGTCATAAAGCCCCC	
CATWTfwd	5′TATGACCTTTTATGATATAGTGGATAGATTTATGATATAATGAGTTATCAACAAATCG	
CATWTrev	5′AGTCTTTTTTTCCGCAGGACGCACTACTC	
AlpBDownfwd	5′GTCCTGCGGAAAAAAAGACTTGAAAAGCC	
AlpBDownrev	5′ACACAGGAAACAGCTATGACTTTACAAAACAAAGTTTTAAACCAC	
CW_003_Intergenic_Left_F	GACGGCCAGTTTGGGGGGTTAAAGTCGC	Complementation, IG::*alpA*
CW_004_Intergenic_Left_R	GCCTTAAATTTAGGCACTTATCCCATAATCTAC	
CW_005_alpA250US_F	TAAGTGCCTAAATTTAAGGCTAATCCCC	
CW_006_alpA250US_R	TTTAAAAAACTTAGAATGAATACCCATAAGAC	
CW_007_ermR_F	ATTCATTCTAAGTTTTTTAAACCCCCTTTCAAAACTAATGC	
CW_008_ermR_R	CTAACCATTCTCCGCAGGACGCACTACTC	
CW_009_Intergenic_Right_F	GTCCTGCGGAGAATGGTTAGGAATAATTTCG	
CW_010_Intergenic_Right_R	CAGCTATGACGACGGATTCTAGTCTTTTAAG	
CW_011_PuC19Vector_F	AGAATCCGTCGTCATAGCTGTTTCCTGTGTG	
CW_012_PuC19Vector_R	AACCCCCCAAACTGGCCGTCGTTTTACAAC	
NF164_G27_igR_F	TATGAAGAGGGGCGTTTGAA	
NF165_G27_igR_R	GCTGGATTACCGTGCGACTG	
alpAU	TAG TAA CGA TGT GTC CGC GA	qRT-PCR
alpAD	AAT TGG TGT TCG TGC CGT AG	
alpBU	AGT AGT GGT AGT GGT GCG AC	
alpBD	CAC TGA GCT GGT TGG AGA GA	
16sF	ATGGATGCTAGTTGTTGGAGGGCT	
16SR	TTAAACCACATGCTCCACCGCTTG	

The Δ*alpA* mutant was constructed by replacing the majority of the *alpA* coding sequence with an *aphA3* (kanamycin resistance) cassette. Briefly, the upstream homologous flanking sequences of *alpA* (containing the first 51 bp of the coding sequence) and the downstream homologous flanking region (containing the last 54 bp) were amplified using primer pairs XL461/XL462 and XL465/XL466, respectively. The *aphA3* cassette was amplified from plasmid pBS-Kan (65) using primers XL463/XL464. These three amplicons were then fused via overlapping extension PCR, and amplified using the external primers XL461 and XL466. The resulting product was transformed into wild-type *H. pylori* G27. Transformants were selected on CHBA plates containing kanamycin, and successful gene replacement was confirmed by PCR amplification and DNA sequencing.

The Δ*alpAB* double mutant was created by replacing the majority of both *alpA-alpB* with an *erm* (erythromycin resistance) cassette. The upstream homologous flanking region (encompassing the first 51 bp of *alpA*) and the downstream homologous flanking region (encompassing the last 48 bp of *alpB*) were amplified with primer pairs XL461/XL468 and XL471/XL472, respectively. The *erm* cassette, including its promoter, was amplified from plasmid pErm-T-tnpR (61) using primers XL469/XL470. These three fragments were fused by overlapping extension PCR and amplified with primers XL461 and XL472. The fusion product was introduced into wild-type *H. pylori* G27, and transformants were selected on erythromycin. Mutants were verified by PCR.

The Δ*alpB::cat* strain was created by replacing the majority of the *alpB* genomic region with a *cat* (chloramphenicol resistance) cassette. The upstream homologous flanking region (encompassing 351 bp upstream of *alpB* and the first 150 bp of *alpB*) and the downstream homologous flanking region (encompassing 351 bp downstream of *alpB* and the last 150 bp of *alpB*) were amplified with primer pairs AlpBUPfwd/AlpBUPrev and AlpBDownfwd/AlpBDownrev, respectively. The *cat* gene was amplified from G27 Δ*flaA::cat* using primers CATWTfwd/CATWTrev. Finally, the backbone, pUC19, was amplified using primer pair pUC19fwd/pUC19rev. These products were fused via Gibson Assembly and then transformed into *E. coli* DH5α. The final plasmid was extracted from *E. coli* DH5α and transformed into *H. pylori* G27. Transformants were selected on chloramphenicol and verified via PCR amplification and sequencing.

The complementation *alpA* (C*alpA*) strain was generated in the Δ*alpA* background (KO2018) using the plasmid pCW001 to place a copy of *alpA* in the intergenic locus used in Langford et al., between G27 genes HPG27_186 and HPG27_187 (66). The upstream and downstream regions were amplified with primers CW03/CW04 and CW09/CW10 (Table 2). Additional fragments containing (i) the *alpA* gene including 250 bp of upstream sequence were amplified with primers CW05/CW06, (ii) an erythromycin resistance cassette (*ermR*) from pEH002 was amplified with primers CW07/CW08, and (iii) the pUC19 plasmid backbone was generated using primers CW11/CW12. All fragments were assembled by NEBuilder HiFi DNA Assembly (NEB) to generate pCW001, which was transformed into chemically competent *E. coli* DH5α. Transformants were screened by colony PCR with primers CW03/CW10 and confirmed by NGS sequencing (Genewiz). The C*alpA* cassette was amplified from pCW001, and transformed into *H. pylori* G27 Δ*alpA* (Table 1), followed by selection on erythromycin 250 µg/mL. Erythromycin-resistant colonies were verified to maintain motility and the correct integration by PCR using primers NF164/NF165.

The Δ*flaA::cat* mutant was constructed by replacing the majority of the *flaA* sequence with the *cat* chloramphenicol resistance gene. Upstream (primers XL118/XL119) and downstream (primers XL122/XL123) were used to amplify regions flanking *flaA*. The *cat* gene (primers XL120/XL121) was used with template pCat-mut. These three amplicons were fused using primers XL118/XL123. To create a Δ*flaA::aphA3* mutant, the *cat* cassette in the Δ*flaA::cat* strain was replaced with *aphA3*. Homologous flanking sequences corresponding to the first and last 250 bp of the *cat* gene (primers XL164/XL165 and XL168/XL169) and the full-length *aphA3* gene (primers XL166/XL167; template *aphA3* cassette) were amplified. These amplicons were fused by SOEing PCR using primers XL168/XL169, and the resulting product was gel purified and transformed into the Δ*flaA::cat* mutant, selecting for kanamycin resistance. The *flaB* allele (Δ*flaB::cat*) was created similarly using primers XL124/XL125 (upstream arm), XL128/XL129 (downstream arm), and XL126/XL127 (*cat* gene) (Table 2).

The Δ*luxS::erm* strain was generated by transforming the wild-type strain with a PCR product containing the *erm* cassette between *luxS* homologous flanking sequences. This PCR product was amplified directly from the genomic DNA of the SS1 Δ*luxS::erm* strain using primers XL294/XL299. The product includes 540 bp upstream of *luxS* (containing the first 40 bp of the coding sequence) and 508 bp downstream (containing the last 8 bp). Transformants were selected on erythromycin and verified by PCR.

### Protein-protein prediction

UCSF’s ChimeraX was used to visualize an AlpA-AlpB complex (67). A structure of each protein was generated by AlphaFold-Multimer (68). The predicted aligned error of the complex was analyzed with ColabFold (69). The maximum residue distance was set to 8 Å, a distance used by other groups modeling proteins (70).

### qRT-PCR

*H. pylori* G27 WT, Δ*alpA*, Δ*alpB*, and Δ*alpAB* strains were grown overnight in BB10, then RNA was extracted using the PureLink RNA Mini Kit (ThermoFisher). Two hundred nanograms of RNA was converted to cDNA using the High-Capacity cDNA Reverse Transcription Kit with RNase Inhibitor (Thermo Fisher Scientific) through a reverse transcription reaction at 25°C for 10 min, 37°C for 2 h, and 85°C for 5 min. For qPCR, 30 ng of converted cDNA was combined with 30 µL of Power SYBR Green PCR Master Mix (Applied Biosystems), 0.25 µM forward primer (alpAU, alpBU, or 16sF), 0.25 µM reverse primer (alpAD, alpBD, or 16SR), and 30 µL water. qPCR amplification was carried out with the following steps: 95°C for 1 min, 45 cycles of 95°C for 15 s and 60°C for 30 s, and 60°C for 1 min. The Cq values of the housekeeping genes were averaged and subtracted from the Cq values of the genes of interest for the ∆Cq measurement. For all samples, a negative no RT control group was included and resulted in Cq values that were undetectable or >8 cycles higher than the +RT sample higher.

### Survival assays

For survival after exposure to antibiotics or serum, *H. pylori* cultures were allowed to form aggregates for 24 h in BB10. They were then checked for aggregation by microscopy and diluted to an OD_600_ of 0.1 at 1 mL in 1.5 mL Eppendorf tubes. For testing antibiotic sensitivity, clarithromycin or metronidazole was added at a final concentration of 15 or 26 μg/mL, respectively. For survival after active serum exposure, cultures were initially diluted to an OD_600_ of 0.2, and then an equal volume of 40% Normal Human Serum (Sigma-Aldrich) or heat-inactivated Normal Human Serum (Sigma-Aldrich) with BB was added to create an OD_600_ of 0.1. The tubes were incubated without shaking under microaerobic conditions for 1.5–2 h. The samples were serially diluted and spread plated on CHBA. Colonies were counted after 4–5 days of growth.

### Crystal violet biofilm assay

*H. pylori* G27 was grown overnight in BB10, then diluted to an OD_600_ of 0.15 in fresh BB10. Two hundred microliters of sample was then added to each well of a sterile polystyrene 96-well plate in triplicate (Costar no. 3370). After static incubation under microaerobic conditions for 3 days, planktonic cells were removed via aspiration, and the plates were washed twice by adding and removing 300 μL 1× PBS per well. Three hundred microliters of crystal violet (0.1% wt/vol) was then added per well and incubated at room temperature for 10 min. The crystal violet was then removed via aspiration, and the wells were once again washed twice using 300 μL 1× PBS per well with removal by aspiration. Wells were air dried, and then 300 μL of 95% ethanol was added to each well and incubated for 10 min at room temperature. The OD_595_ was then measured on a PerkinElmer VICTOR X3 Multimode Plate Reader.
